# Inventory and analysis of literature on the organisation of eight European academic medical centres—A scoping review

**DOI:** 10.1371/journal.pone.0282856

**Published:** 2023-03-10

**Authors:** Ester M. M. Cardinaal, Heleen N. W. Duighuisen, Patrick P. T. Jeurissen, Hubert Berden

**Affiliations:** 1 Operating Rooms, Anesthesiology, Pain and Palliative Medicine, Radboud Universitair Medisch Centrum, Nijmegen, The Netherlands; 2 Radboud Universitair Medisch Centrum, Nijmegen, The Netherlands; 3 Radboud Institute of Health Sciences (RIHS), Nijmegen, The Netherlands; Osun State College of Education, Ila Orangun, NIGERIA

## Abstract

Academic Medical Centres (AMCs) are important organisations for shaping healthcare. The purpose of this scoping review is to understand the scope and type of evidence related to the organisation of European AMCs. We selected the study population intending to obtain a demographic cross-section of European countries: Czech Republic, Germany, Latvia, the Netherlands, Poland, Spain, Sweden and the UK. We focused our search strategy on the relationship between medical schools and AMCs, the organisation of governing bodies, and legal ownership. We searched the bibliographic databases of PubMed and Web of Science (most recent search date 17-06-2022). To enrich the search result, we used Google search engines to conduct targeted searches for relevant websites. Our search strategy yielded 4,672 records for consideration. After screening and reviewing full-text papers, 108 sources were included. Our scoping review provided insight into the scope and type of evidence related to the organisation of European AMCs. Limited literature is available on the organisation of these AMCs. Information from national-level websites complemented the literature and provided a more complete picture of the organisation of European AMCs. We found some meta-level similarities regarding the relationship between universities and AMCs, the role of the dean and the public ownership of the medical school and the AMC. In addition, we found several reasons why a particular organisational and ownership structure was chosen. There is no uniform model for AMC organisations (apart from some meta-level similarities). Based on this study, we cannot explain the diversity in these models. Therefore, further research is needed to explain these variations. For example, by generating a set of hypotheses through in-depth case studies that also focus on the context of AMCs. These hypotheses can then be tested in a larger number of countries.

## 1. Introduction

Academic Medical Centres (AMCs) originated at the end of the 18th century as academic medicine became increasingly institutionalised in a few Paris hospitals. In 1910, the former pedagogue and reformer of the American education system, Abraham Flexner, presented his ideas on the then-current system of medical education in US. This report caused a radical change in the North American medical education system and laid the foundation for today’s evidence-based academic medicine [1, 2]. Although the term academic medical centre is used in numerous countries, there is currently no universally accepted definition. The most common way of defining an AMC is its tripartite mission, which consists of patient care, research, education, and their relation to universities [3–5]. This tripartite mission suggests that AMCs are expected to achieve high standards of (specialised) clinical care, perform fundamental and translational research, and educate doctors and other health professionals [6–8]. The existence of different organisational components within one organisation ensures variability and thus complexity in the organisational structure of an AMC [9]. Since their establishment, these complex organisations have been confronted by healthcare reforms and challenged in their business operations [10, 11]. AMCs are continually adapting and devising solutions to internal and external influences. These include growing medical knowledge, staff shortages, emerging expensive technologies, and ageing patient populations with new demands for care. AMC leaders must cope with tension regarding the distribution of financial resources and competition with other healthcare providers [1, 12–14]. Governments have increasingly emphasised the public and social role of AMCs [15, 16]. According to Raus et. al, AMCs must adapt to this new reality [1]. Although the world around AMCs is changing rapidly, they are still largely organised according to a century old model. However, the current model is slowly evolving towards a dynamic and integrated model in which there is more focus on integration of care and collaboration between different organisations. There is an increasing emphasis on the use of evidence-based medicine and big data and artificial intelligence in clinical practice. To meet contemporary challenges (e.g. financial, human resources, competitive and social), solutions are regularly sought in governance and organisational structures [17–22]. So far, most of the literature on the governance and organisation of AMCs is based on the situation in North America as the issues facing AMCs worldwide are similar [4, 23, 24]. The literature presents little information on AMCs’ governance and organisation in other countries. Our research aims to map and identify literature on the governance and organisation of European AMCs.

Specifically, we identified and analysed the literature on the relationship between medical schools and AMCs, the organisation of governing bodies, and the legal ownership of AMCs in eight European countries: Czech Republic, Germany, Latvia, Netherlands, Poland, Spain, Sweden, and United Kingdom.

## 2. Method

To map and analyse the literature on the governance and organisation of European AMCs, we used a scoping review, a common and valuable approach for mapping and identifying gaps, according to Munn et al. [25]. This review was conducted according to Preferred Reporting Items for Systematic Reviews and Meta-Analysis: for Scoping Reviews (PRISMA-ScR) guidelines [26, 27].

### 2.1. Eligibility criteria

Our study population consisted of a demographic cross-selection of AMC’s from European countries.

We included data from Czech Republic, Germany, Latvia, Netherlands, Poland, Spain, Sweden, and United Kingdom.

### 2.2. Information sources

To identify relevant documents, we searched the following bibliographic databases: PubMed and Web of Science (most recent search date 17-06-2022, see S1 Table). We selected these databases because they include a broad spectrum of scientific, health, and social science journals.

### 2.3. Search strategy

**Fig 1:**
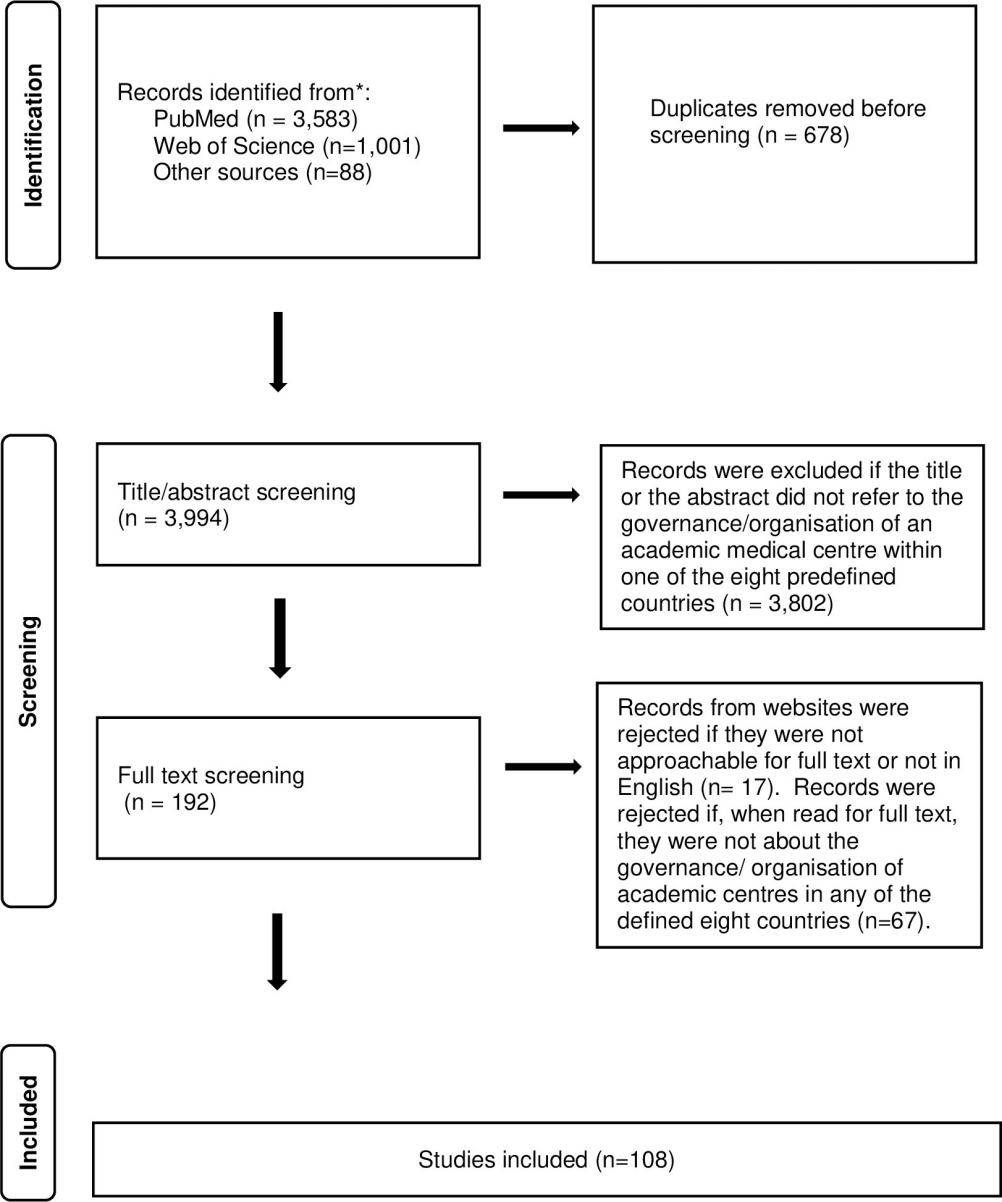
Search results. Identification of studies via databases and other sources.

With the support of a librarian, two authors Ester Cardinaal (EC) and Heleen Duighuisen (HD) prepared a search string for PubMed and Web of Science. S1 Table details the search strategy. The search strategy consisted of three sections: 1) academic medical centres, 2) AMC governance/organisation, and 3) selection of the number of countries. We searched each section using MeSH (Medical Subheadings) terms and title/abstract terms (free text words). Another author Patrick Jeurissen (PJ) verified the search strategy. In total we found 4,584 publications, of which 3,583 were from PubMed and 1,001 were from Web of Science. Fig 1 shows that our search strategy yielded 4,672 records for consideration, of which 4,584 were from database searches and 88 from other sources (see S1 File). After screening and reviewing full-text papers, 108 articles were included.

### 2.4. Selection of sources of evidence

Other sources were included in the review (see S1 File). The review revealed that recent literature on the governance and organisation of the eight European AMCs is limited. Since grey literature is becoming increasingly important for many types of reviews, we have incorporated it to enrich the literature review [28]. The term grey literature is defined as "that which is produced on all levels of government, academics, business and industry in print and electronic formats, but which is not controlled by commercial publishers" [29]. We used Google search engines to conduct targeted searches for websites in the eight countries selected. Government websites and documents were accessed to identify AMCs, research institutes, and medical universities. Subsequently, we screened the websites of identified institutes.

After reviewing the selected literature, we found additional articles using backwards citations and included them in the review. Fig 1 shows the search results.

### 2.5. Critical appraisal, data charting process, synthesis of results

Our literature review included evidence sources from PubMed and Web of Science, as these cover a broad spectrum of scientific, health, and social science journals. The results had to relate to the organisation and governance of AMCs in Czech Republic, Germany, Latvia, Netherlands, Poland, Spain, Sweden, and the United Kingdom. When examining the grey literature, we used the classifications provided by Adams et al.: the degree to which the authority of the literature producer can be determined, and the degree to which literature is published in relation to explicit and transparent criteria [30]. We included sources with high outlet control and credibility, such as government reports, books, journals, and sources with moderate outlet control and credibility, such as annual reports, and news articles. We excluded sources that could be classified as low outlet control and low credibility, such as blogs, tweets, and e-mails [30]. We used Google search engines to access sources that were published on the AMC’s or medical school’s official websites. We selected sources that were related to the governance and/or organisation of the institute.

Given our knowledge of languages, we included sources in English, Dutch and German. We did not include sources in Swedish, Czech, Polish, Spanish, and Latvian unless the websites offered an option for a translation in English.

We examined three elements related to the organisation of an AMC. These elements were drawn from Weiner’s organisational theory [20]. Weiner created eight organisational models based on three dimensions: clinical enterprise organisation, academic-clinical enterprise integration, and authority position of the chief academic officer/medical dean. We used these principles to categorise our findings. One reviewer (HD) developed a data-charting form based on Weiner’s organisation models, which was verified by two other reviewers (EC and PJ) (see Table 1). These three reviewers discussed these results and updated them in an iterative process.

**Table 1:** Overview of the governance and organisation of AMCs per country.

	Czechia	Germany	Latvia	Netherlands	Poland	Spain	Sweden	United Kingdom
**Relation medical school—AMC:**								
Hospital is related to one medical school	X	X		X	X	N/A	X	N/A
Hospital is related to more than one medical school	X		X			N/A		N/A
Medical school owns hospital					X			
**Governing body:**								
One governing body for academic and clinical part		X (integration model)		X	N/A			X (joint leadership + management model)^a^
Separate governing bodies for medical school and AMC	X	X (cooperation model)	X		N/A	X	X	X (joint partnership model)
Medical dean part of clinical governing body		X		X			X	X (joint leadership and management model)
**Legal structure:**								
One legal entity for academic and clinical part		X (integration model)		Xb				
Separate legal entities for academic and clinical part	X	X (cooperation model)	X	X	X	X	X	X
Public ownership^c^	X (central)	X^d^ (regional)	X (central)	X^e^ (central)	X^f^ (central)	X^d^ (regional)	X (regional)	X (central)
Private ownership^c^		X		Xe		X		

N/A: not available

^a^Not used anymore

^b^‘Special’ UMCs

^c^No remark meaning equal ownership for hospital and medical faculty (university)

^d^Most common form of ownership

^e^Hospital and medical school have different legal entities (hospitals private, university 6/8 public 2/8 private)

^f^University hospitals of medical universities^f^

### 2.6. Data items

The variables were limited to eight countries: Czech Republic, Germany, Latvia, Netherlands, Poland, Spain, Sweden, and United Kingdom.

## 3. Results

In this study, institutions are considered AMCs if they are designated with a special term, engaged in patient care, research, and education, and have a relationship with an university (Table 2).

**Table 2:** AMC characteristics per country.

Country	Number of AMCs	AMC per 10 million inhabitants	Local name for AMC	English/literature name for AMC	Tripartite mission		Additional missions
					Simple^a^	Highly complex^b^	
Czechia	10	9.3	Fakultní Nemocnice	University hospital, teaching hospital	X [31]	X [31, 32]	
Germany	34	4.1	Universitätsklinikum	University hospital		X [33, 34]	Innovation [33]
Latvia	3	15.9	Universitātes slimnīca	University hospital		X [35]	Public health [35], innovation [36, 37]
Netherlands	7	4.1	Universitair Medisch Centrum	University Medical Centre		X [38]	Public health, innovation [38]
Poland	N/A	N/A	Szpital Kliniczny	University hospital	X [39, 40]		
Spain	102	21.8	Hospital universitarioc	University hospital	X [41]	X [42]	
Sweden	7	6.9	Universitetssjukhuset	University hospital		X [43–45]	Innovation [46]
United Kingdom	8	1.2	Academic Health Science Centre	Academic Health Science Centre		X [47]	Innovation [48], wealth creation [47]

^a^Simple refers to simple patientcare, research and undergraduate medical education

^b^Highly complex refers to highly complex patientcare, translational research and under- and postgraduate medical education

^c^University- associated hospitals not included

We found meta-level similarities in the relationship between hospitals and medical schools, the role of the dean within governing bodies and legal ownership. However, these similarities have different nuances.

### 3.1. Medical schools and AMCs

In 2017, German medical education was offered at 36 medical faculties at public universities and at a small minority of private universities [49]. Two models are used in the “Universitätsklinikums” [34, 50]. The first is the cooperation model, which is characterised by the legal separation of the university hospital (patient care) and medical school (research and education). The second model is the integration model, in which the medical school (research and education) and university hospital (patient care) form one legal entity and are integrated in all areas [51, 52].

The governance and organisational aspects of the Dutch "Universitair Medisch Centra" are defined in the Dutch Law on Higher Education [53]. The Ministry of Health, Welfare, and Sport and health insurance companies contribute to financing patient care; while the Ministry of Education, Culture, and Science finance education and research. Universities contribute to the financing of education and research, as well as providing a workplace to train doctors in medical schools and hospitals.

In Poland, the Ministry of Health has established medical universities, which in turn have established medical university hospitals. The Ministry of Health and territorial self-governments are responsible for the governance of the health system, medical research, and education [54]. University hospitals are specialised hospitals that provide highly complex patient care, as well as perform medical education and research via the medical school [55, 56]. The total number of Polish AMCs is difficult to establish due to various data sources. There is a variance from nine medical universities [57] to fifteen medical faculties [58] and up to 20 medical faculties (public and private, including twelve medical universities). In 2016, medical universities owned 36 hospitals [54]. Medical education used to only be offered at medical universities. However, as a result of physician shortages, usually in provinces that did not count any medical universities, medical education was also offered at medical faculties of nonmedical, multi-faculty universities [54]. Medical universities use various non-academic hospitals for their clinical training.

In Spain, there are 102 Spanish “hospitales universitarios” (university or teaching hospitals) [59]. The relationship between Spanish teaching hospitals and universities is defined by the teaching hospital title granted by the government if most or all the hospital’s care units are used for either clinical teaching or university research.

In Sweden, the tripartite mission is present in seven university hospitals (“universitetssjukhuset”) that provide highly specialised care [43] and fulfil a role in the training of medical staff [46]. At least until 2011, academic missions were fulfilled by seven public universities providing undergraduate medical education [60].

In 2007, the United Kingdom’s Department of Health established several cross-sector collaborations, one of which was the establishment of academic health science centres [61]. Partnerships consist of universities, medical schools [62], and hospitals, which are known as either university or teaching hospitals.

In the Czech Republic, the tripartite mission can be seen in so-called university hospitals or teaching hospitals (“Fakultní Nemocnice”) [32]. All university hospitals have partnerships with medical faculties [63].

In Latvia, clinical education takes place at three university hospitals (“universitātes slimnīca”) and non-university hospitals [35].

### 3.2. Governing bodies

Although the organisation of European AMCs differs, the role of the dean is important both in the medical school and hospital, confirming the findings of Weiner et al. [20].

Within the different contextual frameworks, these European countries have shaped the governing bodies of an AMC in their own ways.

For example, in Germany, hospital boards (“Klinikumsvorstand”) head university hospitals. In general, the board includes a medical director (often the chairperson), administrative/commercial director, nursing director, and medical dean. Tasks within the boards may differ and depend on the organisational model, legal form, and board composition. The dean is responsible for academic matters such as research and teaching. These tasks are similar across all German university hospitals [50].

Dutch AMCs and medical faculties delegate responsibilities to an AMC executive board to ensure effective fulfilment of the tripartite mission of clinical care, medical education and research. For the same reason, the dean has a role in both the medical school and AMC boards [4]. The administration and working relationship between the university and hospital are regulated by law [53]. Members of the supervisory board are appointed by the Minister of Culture and Education [4, 64].

Swedish AMCs are governed by the respective regional administrative entities [65]. University hospitals can be subordinate to a regional administrative body that manages other hospitals [66]. The dean is the highest governing authority of the medical school [67] and is subordinate to the vice-chancellor [68–70] who is in turn subordinate to the university board, the highest governing body of the university [67, 69, 71]. Most of the board is appointed by the government according to Swedish law [72]. In some cases, the university hospital boards include the dean [73] and, others do not [74, 75].

In the United Kingdom, all university hospitals are part of the National Health Service (NHS). These hospitals are registered with either NHS Trusts or NHS Foundation Trusts [76]. NHS Trusts are statutory organisations that have been authorised by the Secretary of State for Health and Social Care to operate care centres or hospitals. NHS Foundation Trusts are more autonomous organisations with boards accountable to a board of governors that represents local communities [77].

In the Czech Republic, the governing bodies of medical schools typically consist of a dean as the highest governing body of the medical school, faculty management, an academic senate, and a scientific board [78–81]. The academic senate and scientific board also have important decision-making powers [82]. The Ministry of Education supervises educational tasks [32].

Latvian AMC governance is defined in Latvian law, which provides for a two-tier structure of a management/executive board and supervisory board [83, 84]. The university hospital’s executive board is the primary governing body within the university hospital that falls under the supervisory board. The nomination of members for both boards is also regulated by law. The highest governing body of the medical school in Latvia is the vice-rector or dean (subordinate to the vice-rector) [85]. The vice-rector is subordinate to the rector of the university [86].

In Poland, university hospitals are supervised by the Ministry of Health [56] with a management team headed by a director or CEO (Chief Executive Officer) [87, 88]. Within medical universities, the highest governing body is the rector [89–91] with the dean of the medical school governing the medical school [40, 92]. The vice-rector is responsible for clinical affairs, such as the supervision, inspection, and administrative support of teaching hospitals [39, 89, 93].

In Spain, the highest governing body within the university hospital is the management (“Gerencia”) [94, 95] while the faculty board is the highest governing body within the medical school. Both the university hospital and medical school are governed by the faculty board and management team [96–98]. The dean plays an important role at both the university hospital and medical school [96, 97, 99].

### 3.3. Legal ownership

Public ownership of medical schools and associated university hospitals is the most common form of ownership. Most national models show separate legal entities, suggesting that functional integration is the preferred model over institutional integration. Countries without an integration model described in the literature show characteristics of functional integration in a "collaborative" or "joint partnership" model. These models exhibit separate governing bodies and legal entities for both hospitals and medical schools. This observation has a wide range of differences as illustrated by numerous country-specific nuances.

For instance, Czech university hospitals have partnerships with medical faculties. University hospitals can provide clinical education for several medical faculties [31].

In Germany, university hospitals can be regarded as a network that creates collaboration between patient care, research, and education, although they are not obliged to perform research or provide education [34]. Most university hospitals have become independent legal institutions under public law. Some German states have implemented the “corporate solution” in which the university hospital operates in the form of a corporation under public law and, provides members of the corporation with a certain degree of influence. Conversely a minority of the states have arranged university hospitals under private law. A minority of university hospitals operates under other legal forms [50].

In Latvia, universities and university hospitals are state-owned [35]. This is in contrast to most Latvian hospitals, which are owned by municipalities and have less stringent operating obligations than state-owned hospitals [100].

The Dutch Law on Higher Education views universities and AMCs as separate and independently governed units. The law regulates the administration and working relationship between universities and hospital [53]. Of the eight Dutch AMCs six involve partnerships between public universities and private hospitals where both the academic and clinical parts maintain their own legal entity. The remaining two “special” AMCs involve partnerships between private universities and hospitals [4, 64].

In Poland, university hospitals are owned by medical universities, while medical universities are publicly owned [55].

Spain is divided into seventeen autonomous communities (ACs), each with its own legislative and executive autonomy, parliament, and government. The decentralisation of public and social security healthcare centres, services, and competencies in 2002 led to an increase in regulative powers for the ACs [101]. ACs lead public health services via local public agencies. Although ownership and organisational models of hospital care vary substantially, most university hospitals are owned by these local public agencies [101]. Private non-profit ownership plays a substantial role in the governance of the Madrid and Catalonia ACs [59]. Several university hospitals are affiliated with a medical school. Partnerships between medical faculties, university hospitals (or university-associated hospitals), and medical faculties and research institutes ensure clinical and research education [102, 103]. Currently, there are 49 faculties where medical degrees can be obtained, with the majority of them being owned by public governments through ACs [104].

In the United Kingdom, all university hospitals are part of the NHS [61, 62]. Partnerships consist of universities and hospitals, which are called either universities or teaching hospitals. Both universities and hospitals are publicly owned [62]. Additionally, instead of being subject to the Department of Health, foundation trusts are supervised by Monitor, a regulatory body [77]. Accountability is divided since universities, university hospitals and, research institutes are accountable to different government departments [47, 105].

The Swedish healthcare system is decentralised, with the main responsibility for the provision and financing of healthcare resting with regional bodies. There are 21 counties, which are further divided into six medical regions to provide better cooperation in tertiary care [44]. University hospitals, like most other hospitals in the country, http://eprints.lse.ac.uk/43952/1/Sweden health system review (lsero).pdfare publicly owned [43].

## 4. Discussion

Differences in the organisation, governing bodies, and legal structures of European AMCs have a cultural and historical background in which the government’s views, laws, and regulations play an important role. At a meta-level, this study identified three common factors across the eight countries studied. First, most countries operate with separate governing bodies and legal entities for the medical school and hospital. Second, most countries have a dean who simultaneously plays a role in the organisation of both the medical school and hospital. Finally, most countries prefer a functionally integrated relationship between medical schools and hospitals. Despite these common factors, during our research, we came across a variety of reasons why a particular governance organisation and ownership structure was chosen. A multitude of internal and external conditions, challenges, and objectives drive organisations to rethink and adapt their organisation and legal structures [11, 12, 106]. Examples from our study illustrate these different national perspectives and circumstances.

Since the 1990s, Germany has wanted to measure up in global competition. To achieve this, the country needed to become more manageable and flexible [34, 107, 108]. In 2006, the privatisation of two merged university hospitals in Germany was conducted to achieve synergy effects, but ultimately failed [109, 110]. For the same reason, one of the Dutch AMCs recently changed the university and medical school to two different legal entities [111]. Similar considerations in the United Kingdom led to the formation of academic health science centres in 2009, building on existing AMC structures [23, 112]. In Poland, there were other reasons for opting to separate universities and AMCs. Medical faculties were part of universities until the 1950s. They became independent medical universities to make them more accessible to the public [113]. Moreover, due to a shortage of doctors, mostly in provinces that did not have medical universities, the government had to allow nonmedical universities to train doctors as well [56]. In Sweden, collaborations and mergers between AMCs and other hospitals, universities, and training institutions are increasing, mainly to meet steadily rising costs [60, 114].

Weiner et al. [20] presented eight organisational models based on three dimensions: clinical enterprise organisation, academic-clinical enterprise integration, and the authority position of the chief academic officer. The eight models describe mutual relationships and propose ideas on the distribution of power in decision-making processes. However, Weiner et al. emphasise that “few, if any [of the existing AMCs], are likely to resemble these pure forms”, demonstrating the complexity and variability of an AMC organisation. This variability is reflected in our results. Therefore, it is striking that despite this multitude of different perspectives, more or less the same organisation, legal structure, and functionally integrated relationship between universities and hospitals seem to be preferred without considering the challenges faced by AMCs. Our review reveals that while the baseline organisation of AMCs seems similar, there is significant variation in how they are implemented in practice. Factors other than organisation are more important in determining the functionality of AMCs.

Our research was deliberately broad in scope to get an overview of the range of literature on AMC governance and organisation. Despite its strengths, this review has several limitations. We are aware that some literature may not have been detected as we only consulted two databases. We conducted manual searches for additional information. The definition of an AMC is not unambiguous and depends on contextual factors; thus, it may not be possible to find all relevant organisations. We addressed this by establishing a definition for an AMC beforehand (university-AMC relationship and tripartite mission). Furthermore, we acknowledge that the selection of only eight European countries may not reflect the diversity of AMC governance and organisation in other European countries. Finally, we note the limitation that the purpose of scoping reviews is not to produce a critically assessed and synthesised answer to a particular question, but to provide an overview of the evidence; therefore, its practical implications are quite different from those of a systematic review. Despite these limitations, we believe that this study identified evidence-based gaps, providing a stimulus to fill those gaps through further research.

## 5. Conclusion

Little literature exists on the organisation of European AMCs. The use of national-level websites complements the literature and gives a more complete picture of the organisation of these organisations. The organisation of AMCs in the eight countries studied show meta-level similarities in terms of the relationship between universities and AMCs, the role of the dean and the public ownership of the medical school and the AMC. Most countries have separate governing bodies and legal entities for the medical school and the hospital, have a dean who simultaneously plays a role in the organisation of both the medical school and the hospital, and prefer a functionally integrated relationship between the medical school and the hospital. However, the organisation of AMCs in the eight countries seems to differ when it comes to why a particular organisation and ownership structure is chosen. Several factors influence the choice of a particular organisation and legal structure including internal and external circumstances, challenges and objectives. There is no uniform model for AMC organisations (apart from some meta-level similarities). Based on this study, we cannot explain the diversity in these models. Therefore, further research is needed to explain these variations. For example, by generating a set of hypotheses through in-depth case studies that also focus on the context of AMCs. These hypotheses can then be tested in a larger number of countries.

## Supporting information

S1 Checklist (PRISMA-ScR) checklist. (DOCX)

S1 Table Search details and history. (DOCX)

S1 File Other sources. (DOCX)
