# Supplementary material for: Inventory and analysis of literature on the organisation of eight European academic medical centres—A scoping review
Source: PLoS One. 2023 Mar 10;18(3):e0282856. doi: 10.1371/journal.pone.0282856 (PMC10004499; doi:10.1371/journal.pone.0282856)
Supplement: S1 Table — (DOCX) [file pone.0282856.s002.docx]

# S1_Table 1_Search details and history

PubMed search June 17, 2022: 3,583 records

Web of Science search June 17, 2022: 1,001 records

## The search strategy consisted of three sections: 1) academic medical centres 2) their governance/organisation 3) selection of number of countries. Each section was searched using MeSH terms and title/abstract terms (free text words). The Web of Science search strategy was constructed similar to the PubMed search. In Web of Science, the topic field (TS) was searched as it searches the title, abstract, author keywords and Keywords Plus.

## PubMed search

Date searched: June 17, 2022

| **Search** | **Query** | **Results** |
| --- | --- | --- |
| #4 | #1 AND #2 AND #3 | 3,583 |
| #3 | ("Poland"[Mesh] OR "Latvia"[Mesh] OR "Czech Republic"[Mesh] OR "Netherlands"[Mesh] OR "Germany"[Mesh] OR "Spain"[Mesh] OR "Sweden"[Mesh] OR "United Kingdom"[Mesh] OR Poland[tiab] OR Latvia[tiab] OR Czech Republic[tiab] OR Czechia [tiab] OR Netherlands[tiab] OR Germany[tiab] OR Spain[tiab] OR Sweden[tiab] OR United Kingdom[tiab] OR UK[tiab] OR Great Britain[tiab]) | 1,006,241 |
| #2 | ("Governing Board"[Mesh:NoExp] OR "Models, Organizational"[Mesh] OR "Academic Medical Centers/organization and administration"[Mesh] OR Govern*[tiab] OR Tripartite Mission*[tiab] OR Organisation*[tiab] OR Organization*[tiab] OR Dean*[tiab]) | 754,199 |
| #1 | ("Academic Medical Centers"[Mesh] OR Academic Medical Cent*[tiab] OR Academic Health Cent*[tiab] OR Academic Health Science Cent*[tiab] OR Academic Health Science*[tiab] OR Teaching Hospital*[tiab] OR Medical School*[tiab] OR Tertiary Hospital*[tiab] OR Clinical Enterprise*[tiab] OR University Hospital*[tiab] OR Referral Hospital* [tiab] OR University Medical Cent*[tiab]) | 296,647 |

## Web of Science

Date searched: June 17, 2022

Web of Science Core Collection consists of the following indices:

- Science Citation Index Expanded (SCI-EXPANDED)--1945-present
- Social Sciences Citation Index (SSCI)--1956-present
- Arts & Humanities Citation Index (AHCI)--1975-present
- Conference Proceedings Citation Index – Science (CPCI-S)--1999-present
- Conference Proceedings Citation Index – Social Science & Humanities (CPCI-SSH)--1999-present
- Book Citation Index – Science (BKCI-S)--2005-present
- Book Citation Index – Social Sciences & Humanities (BKCI-SSH)--2005-present
- Emerging Sources Citation Index (ESCI)--2005-present
- Current Chemical Reactions (CCR-EXPANDED)--1985-present
- Index Chemicus (IC)--1993-present

| **Search** | **Query** | **Results** |
| --- | --- | --- |
| #4 | #1 AND #2 AND #3 | 1,001 |
| #3 | TS=(Poland OR Latvia OR Czech Republic OR Czechia OR Netherlands OR Germany OR Spain OR Sweden OR United Kingdom OR UK OR Great Britain) | 1,135,630 |
| #2 | TS=(Govern* OR Tripartite Mission* OR Organisation* OR Organization* OR Dean*) | 2,197,570 |
| #1 | TS=(“Academic Medical Cent*” OR “Academic Health Cent*” OR “Academic Health Science Cent*” OR “Academic Health Science*” OR “Teaching Hospital*” OR “Medical School*” OR “Tertiary Hospital*” OR “Clinical Enterprise*” OR “University Hospital*” OR “Referral Hospital*” OR “University Medical Cent*”) | 229,633 |
