# Supplementary material for: Inventory and analysis of literature on the organisation of eight European academic medical centres—A scoping review
Source: PLoS One. 2023 Mar 10;18(3):e0282856. doi: 10.1371/journal.pone.0282856 (PMC10004499; doi:10.1371/journal.pone.0282856)
Supplement: S1 File — (DOCX) [file pone.0282856.s003.docx]

S1_File_Other_Sources

1. Raus K, Mortier E, Eeckloo K. Past, present and future of university hospitals. Acta Clin Belg. 2020;75(3):177-84. Raus K, Mortier E, Eeckloo K. Past, present and future of University Hospitals. Acta Clinica Belgica. 2019;75(3):177–84.
2. Lawrence Diller. 100 years later, the Flexner Report is still relevant. Hastings Center Report. 2010;40(5):5–.
3. Blumenthal D, Meyer GS. The future of the academic medical center under Health Care Reform. New England Journal of Medicine. 1993;329(24):1812–4.
4. Dzau VJ, Cho A, ElLaissi W, Yoediono Z, Sangvai D, Shah B, et al. Transforming Academic Health Centers for an uncertain future. New England Journal of Medicine. 2013;369(11):991–3.
5. Wartman SA. Toward a virtuous cycle: The changing face of Academic Health Centers. Academic Medicine. 2008;83(9):797–9.
6. Guo KL. Roles of managers in academic health centers. The Health Care Manager. 2002;20(3):43–58.
7. Hastings DA, Crispell KR. Policy-making and governance in academic health centers. Academic Medicine. 1980;55(4):325–32.
8. Lee PR. A tiger by the tail. Academic Medicine. 1973;48(1):27–39.
9. Durán Antonio, Wright S. Understanding hospitals in Changing Health Systems. Basingstoke: Palgrave Macmillan; 2021.
10. Chari R, O’Hanlon C, Chen P, Leuschner K, Nelson C. Governing Academic Medical Center Systems. Academic Medicine. 2018;93(2):192–8.
11. Ministerie van Onderwijs, Cultuur en Wetenschap. Kamerbrief over Positioneringsnota UMC's [Internet]. Platform open overheidsinformatie. 2014 Jul [cited 2022 Oct 29]. Available from: <https://open-pilot.overheid.nl/Details/ronl-archief-36d43656-fc9b-4073-9c20-dc3bdc308837/1?hit=4&sort=date-asc&count=50&informatiesoort_filter=&beschikbaar_filter=ouder-dan-een-jaar&thema=c_d0463fb7&organisatie=mnre1109>
12. Woolf SH. The meaning of translational research and why it matters. JAMA. 2008;299(2).
13. Minkman MMN. Longing for Integrated Care: The Importance of effective governance. International Journal of Integrated Care. 2017;17(4).
14. Weiner BJ, Culbertson R, Jones RF, Dickler R. Organizational models for Medical School—Clinical Enterprise Relationships. Academic Medicine. 2001;76(2):113–24.
15. Schreyögg J, von Reitzenstein C. Strategic groups and performance differences among Academic Medical Centers. Health Care Management Review. 2008;33(3):225–33.
16. Hauge M. About US - University Hospital in Motol [Internet]. Fakultní nemocnice v Motole. c2022 [cited 2022 Oct 30]. Available from: <https://www.fnmotol.cz/en/o-nas/>
17. Alexa J, Rečka L, Votápková J, Van Ginneken E, Spranger A, Wittenbecher F. The performance of the Czech health system: Untapped potential for efficiency gains and health improvement. European Journal of Public Health. 2015;25(suppl_3).
18. Die Sonderrolle der deutschen Universitätsklinika [Internet]. Die Deutschen Universitätsklinika. [cited 2022 Oct 31]. Available from: <https://www.uniklinika.de/die-deutschenuniversitaetsklinika/sonderrolle-universitaetsmedizin/>
19. Deutscher Bundestag WD. Ausarbeitung Begriff, Rechtsformen und Finanzierung der Universitätskliniken in Deutschland [Internet]. 2009 [cited 2022 Oct 30]. Available from: <https://www.bundestag.de/resource/blob/417706/6e9eb8e17dbe2a1905d3fc8d9a4acf51/WD-9-007-16-pdf-data.pdf>
20. Public Report on the State-Owned Enterprises and Shares in 2018 [Internet]. Valsts Kapitālsabiedrību Pārvaldība. Cross-Sectoral Coordination Centre Republic of Latvia; 2019 [cited 2022 Oct 30]. Available from: <https://www.valstskapitals.gov.lv/images/userfiles/Latvia_Report-on-State-Owned-Enterprises-and-Shares-in-2018_ENG_.pdf>
21. Paula Stradiņa Klīniskā universitātes slimnīca. Mission / Vision / Strategy [Internet]. Stradiņa slimnīca. 2019 [cited 2022 Oct 30]. Available from: <https://www.stradini.lv/lv/content/misija-vizija-strategija>
22. Rīgas Austrumu Klīniskā Univiersitātes Slimnīca. About Riga East University Hospital [Internet]. aSlimnīca. 2022 [cited 2022 Oct 30]. Available from: <https://aslimnica.lv/en/>
23. Nederlandse Federatie van Universitair Medische Centra. Over de NFU [Internet]. NFU. 2022 [cited 2022 Oct 30]. Available from: <https://www.nfu.nl/nfu/over-de-nfu>
24. Medical University of Lodz. Teaching hospitals [Internet]. 2022 [cited 2022 Oct 30]. Available from: <https://studymed.umed.pl/about-mul-2/teaching-hospitals/>
25. Medical University of Warsaw. Teaching hospitals [Internet]. [cited 2022 Oct 30]. Available from: <https://www.wum.edu.pl/en/about-university/teaching-hospitals>
26. Puerta JL, Martín-Moreno JM, Bravo S, Gutiérrez-Fuentes JA. Valoración de la investigación que se realiza en los hospitales españoles. Revista Clínica Española. 2011;211(4):169–78.
27. Agencia Estatal Boletín Oficial del Estado. Consolidated Legislation [Internet]. 2021 [cited 2022 Oct 30]. Available from: <https://www.boe.es/buscar/act.php?id=BOE-A-1986-20584>
28. OECD and European Observatory on Health Systems and Policies. Sweden: Country health profile 2021. State of Health in the EU. 2021;
29. Anell A, Glenngård AH, Merkur S. Sweden health system review. Health Syst Transit. 2012;14(5):1-159. PMID: 22894859
30. Commonwealth Fund. International Profiles of Health Care Systems, 2015 [Internet]. 2016 [cited 2022 Oct 30]. Available from: <https://www.commonwealthfund.org/publications/fund-reports/2016/jan/international-profiles-health-care-systems-2015>
31. Universitetssjukhuset Örebro. [Internet]. About Us. 2014 [cited 2022 Oct 30]. Available from: <https://usorebro.se/en/About-us/>
32. The AHSN Network. About academic health science networks [Internet]. 2019 [cited 2022 Oct 30]. Available from: <https://www.ahsnnetwork.com/about-academic-health-science-networks>
33. Zavlin D, Jubbal KT, Noé JG, Gansbacher B. A comparison of medical education in Germany and the United States: from applying to medical school to the beginnings of residency. Ger Med Sci. 2017 Sep 25;15:Doc15. doi: 10.3205/000256. PMID: 29051721; PMCID: PMC5617919.
34. Wettenbank. Wet op het hoger onderwijs en wetenschappelijk onderzoek [Internet]. 2022 [cited 2022 Oct 30]. Available from: <https://wetten.overheid.nl/BWBR0005682/>
35. Sowada C, Sagan A, Kowalska-Bobko I, Badora-Musial K, Bochenek T, Domagala A, et al. Poland: Health System Review. Health Syst Transit. 2019 Jun;21(1):1-234. PMID: 31333192.
36. Dubas-Jakóbczyk K, Kocot E, Kozieł A. Financial Performance of Public Hospitals: A cross-sectional study among Polish providers. International Journal of Environmental Research and Public Health. 2020;17(7):2188.
37. OECD and European Observatory on Health Systems and Policies. Poland: Country health profile 2019: En [Internet]. OECD. [cited 2022 Nov 1]. Available from: <https://www.oecd.org/health/poland-country-health-profile-2019-297e4b92-en.htm>
38. European Statistical System. Statistics Poland [Internet]. [cited 2022 Nov 1]. Available from: <https://stat.gov.pl/en/topics/statistical-yearbooks>
39. The Polish National Agency for Academic Exchange. The Polish National Agency for Academic Exchange [Internet]. NAWA. 2022 [cited 2022 Nov 2]. Available from: <https://nawa.gov.pl/en/nawa>
40. Ministry of Health. National Catalogue of Hospitals [Internet]. [cited 2022 Oct 30]. Available from: <https://www.mscbs.gob.es/ciudadanos/prestaciones/centrosServiciosSNS/hospitales>
41. Lindgren S, Brännström T, Hanse E, Ledin T, Nilsson G, Sandler S, et al.; Medical education in Sweden [Internet]. Medical teacher. U.S. National Library of Medicine; [cited 2022 Oct 31]. Available from: <https://pubmed.ncbi.nlm.nih.gov/21942478/>
42. Medical Schools Council. Medical schools [Internet]. 2018 [cited 2022 Oct 30]. Available from: <https://www.medschools.ac.uk/studying-medicine/medical-schools>
43. Medical Study Guide. Study medicine in the Czech Republic [Internet]. [cited 2022 Oct 31]. Available from: <https://medicalstudyguide.com/medicine-in-czech-republic.html>
44. Vrije Universiteit Amsterdam. Policy and organisation [Internet]. [cited 2022 Oct 30]. Available from: <https://vu.nl/en/employee/policy-and-organisation>
45. Region Östergötland. Our hospitals [Internet]. Healthcare about the region. 2019 [cited 2022 Oct 30]. Available from: <https://www.regionostergotland.se/Halsa-och-vard/Vara-sjukhus/>
46. Skåne University Hospital. Our organisation [Internet]. [cited 2022 Oct 30]. Available from: <https://vard.skane.se/en/skane-university-hospital/about-us/our-organisation/>
47. Umea University. About the faculty. [cited 2022 Oct 30]. Available from: <https://www.umu.se/en/faculty-of-medicine/about-the-department/>
48. Linköping University. The organisation of Linköping University [Internet]. [cited 2022 Oct 30]. Available from: <https://liu.se/en/about-liu/organisation>
49. Lund University. Organisational structure [Internet]. [cited 2022 Oct 30]. Available from: <https://www.lunduniversity.lu.se/about-lund-university/management-leadership/organisational-structure>
50. Örebro University. Organisation and governance [Internet]. [cited 2022 Oct 30]. Available from: <https://www.oru.se/english/about-us/organisation-and-governance/>
51. Uppsala University. The University Board [Internet]. [cited 2022 Oct 30]. Available from: <https://www.uu.se/en/about-uu/organisation/university-board/>
52. Swedish Council for Higher Education. The Swedish Higher Education Act (1992:1434) [Internet]. UHR.se. 2022 [cited 2022 Oct 30]. Available from: <https://www.uhr.se/en/start/laws-and-regulations/Laws-and-regulations/The-Swedish-Higher-Education-Act/>
53. Region Östergötland. About the region. Healthcare. [Internet]. Hospital Management Linköping. 2019 [cited 2022 Oct 30]. Available from: <https://www.regionostergotland.se/Halsa-och-vard/Vara-sjukhus/Universitetssjukhuset-i-Linkoping/Sjukhusledning-Linkoping/>
54. Karolinska Institutet. Board of Karolinska Institutet [Internet]. [cited 2022 Oct 30]. Available from: <https://ki.se/en/about/board-of-karolinska-institutet>
55. Akademiska Sjukhuset. [Internet]. Welcome to Uppsala University Hospital. [cited 2022 Oct 30]. Available from: <https://www.akademiska.se/en/>
56. Palacký University Olomouc. Faculty bodies [Internet]. Faculty of Medicine and Dentistry. 2022 [cited 2022 Nov 1]. Available from: <https://www.lf.upol.cz/en/about/faculty-bodies/>
57. Charles University Third Faculty of Medicine. Statutory bodies [Internet]. 2022 [cited 2022 Nov 1]. Available from: <https://www.lf3.cuni.cz/3LFEN-155.html>
58. Charles University. Organization structure [Internet]. 2013 [cited 2022 Nov 1]. Available from: <https://www.lfhk.cuni.cz/Faculty/Organization-structure/>
59. University Masaryk. Faculty authorities [Internet]. Faculty of Medicine Masaryk University | MED MUNI. [cited 2022 Oct 30]. Available from: <https://www.med.muni.cz/en/about-faculty/faculty-authorities>
60. Charles University First Faculty of Medicine. Faculty management [Internet]. First Faculty of Medicine, Charles University. [cited 2022 Nov 1]. Available from: <http://en.lf1.cuni.cz/faculty-management>
61. Bērnu klīniskā universitātes slimnīca. Scheme of the structure of the hospital [Internet]. 2018 [cited 2022 Nov 1]. Available from: <https://www.bkus.lv/lv/content/slimnicas-strukturas-shema>
62. University of Latvia. [Internet]. University structure. 2022 [cited 2022 Oct 31]. Available from: <https://www.lu.lv/en/about-us/structure/university-structure/>
63. Rīga Stradiņš University. Structure [Internet]. RSU. [cited 2022 Oct 30]. Available from: <https://www.rsu.lv/en/about-us/structure>
64. Sobre la Universidad Pompeu Fabra [Internet]. Estructura y organización - Sobre la Universidad Pompeu Fabra (UPF). [cited 2022 Oct 30]. Available from: <https://www.upf.edu/es/web/universitat/estructura-organitzacio>
65. Independent Clinical Hospital no. 1 Lublin. Directorate SPSK No. 1 [Internet]. <https://www.spsk1.lublin.pl/dyrekcja>. [cited 2022 Oct 30]. Available from: <https://www.spsk1.lublin.pl/dyrekcja>
66. Pomorski Uniwersytet Medyczny W Szczecinie. [Internet]. Clinical Hospital. 2017 [cited 2022 Oct 30]. Available from: <https://old.pum.edu.pl/administracja/biuro-ds.-szpitali-klinicznych>
67. Poznan University of Medical Science | Center for Medical Education. Authorities [Internet]. [cited 2022 Oct 30]. Available from: <https://pums.ump.edu.pl/contact/authorities.html>
68. Wroclaw Medical University. Authorities [Internet]. 2021 [cited 2022 Oct 30]. Available from: <https://www.en.umed.wroc.pl/about/authorities>
69. Medical University of Gdańsk. Directory of MUG organizational structure [Internet]. 2022 [cited 2022 Nov 2]. Available from: <https://structure.mug.edu.pl/>
70. Medical University of Warsaw. Teaching hospitals [Internet]. 2021 [cited 2022 Oct 30]. Available from: <https://www.wum.edu.pl/en/about-university/teaching-hospitals>
71. Medical University of Lodz. Teaching hospitals [Internet]. 2022 [cited 2022 Oct 30]. Available from: <https://studymed.umed.pl/about-mul-2/teaching-hospitals/>
72. Medical University of Lublin. Organizational structure [Internet]. 2014 [cited 2022 Oct 30]. Available from: <https://www.umlub.pl/en/university/organizational-structure/>
73. Vall d'Hebron Hospital. Management [Internet]. [cited 2022 Oct 30]. Available from: <https://hospital.vallhebron.com/es/el-hospital/organizacion-y-participacion/equipo-directivo>
74. University Hospital Virgen de las Nieves. Organization [Internet]. 2022 [cited 2022 Oct 30]. Available from: <https://www.huvn.es/el_hospital/organizacion>
75. Universidad Complutenze Madrid. Composition of the Faculty Board [Internet]. Portada. [cited 2022 Nov 2]. Available from: <https://medicina.ucm.es/composicion-de-la-junta-de-facultad>
76. Universidad Pompeu Fabra. Structure and organization [Internet]. Estructura y organización - Sobre la Universidad Pompeu Fabra (UPF). [cited 2022 Oct 30]. Available from: <https://www.upf.edu/es/web/universitat/estructura-organitzacio>
77. Universidad de Granada. Governing bodies [Internet]. Faculty of Medicine. [cited 2022 Oct 30]. Available from: <https://medicina.ugr.es/facultad/organos-de-gobierno#title1>
78. Universitat de Girona. Management team [Internet]. [cited 2022 Nov 2]. Available from: <https://www.udg.edu/es/fm/facultat/estructura/organs-de-govern/equip-de-direccio>
79. OECD/European Observatory on Health Systems and Policies (2021). Latvia: Country Health Profile 2021 [Internet]. [cited 2022 Nov 2]. Available from: <https://doi.org/10.1787/919f55f0-en>
80. Bernal-Delgado E, Garcia-Armesto S, Oliva J, Sanchez Martinez FI, Repullo JR, Pena-Longobardo LM, et al. Spain: Health System Review. Health Syst Transit. 2018 May;20(2):1-179. PMID: 30277216.
81. Universidad autónoma de Madrid. Research Centers and Institutes [Internet]. [cited 2022 Nov 2]. Available from: <https://www.uam.es/Medicina/CentrosInvestigacion/1242658546467.htm?language=es&nodepath=Centros%20e%20Institutos%20de%20Investigaci?n>
82. Universidad de Sevilla. Teaching organisation [Internet]. Organización docente | Ordenación Académica :: Universidad de Sevilla. [cited 2022 Nov 2]. Available from: <http://servicio.us.es/academica/organizacion-docente>
83. Ministerio de Educacion, Cultura y Deporte. Qu estudiar y dnde en la Universidad (QEDU) [Internet]. [cited 2022 Nov 2]. Available from: <https://www.educacion.gob.es/notasdecorte/compBdDo>
84. Academic Health Centers: Leading Change in the 21st Century. Academic Emergency Medicine. 2004;11(7):802–6.
85. Arbeitsgemeinschaft Hochschulmedizin. Privatisierung der Hochschulmedizin Gescheitert [Internet]. 2013 [cited 2022 Nov 2]. Available from: <http://www.ag-hochschulmedizin.de/files/2013/Resolution-Privatisierung_der_Hochschulmedizin_7-2013.pdf>
86. Radboud Universitair Medisch Centrum. Ontvlechting een feit en Nieuwe Raad van Toezicht [Internet]. [cited 2022 Oct 30]. Available from: <https://www.radboudumc.nl/nieuws/2021/ontvlechting-een-feit-en-nieuwe-raad-van-toezicht#:~:text=Per%201%20januari%202021%20is,het%20huidige%20college%20van%20bestuur>
87. Department of Health and Social Care. High quality care for all: NHS Next Stage Review Final Report [Internet]. GOV.UK. GOV.UK; 2008 [cited 2022 Oct 30]. Available from: <https://www.gov.uk/government/publications/high-quality-care-for-all-nhs-next-stage-review-final-report>
88. Kostrzewski J. An integrated approach to health services and Manpower Development: The experience of Poland. Health Policy and Education. 1980;1(2):197–211.
